# Prevalence of Osteoporosis and Its Risk Factors in Men with COPD in Qazvin

**DOI:** 10.1155/2016/4038530

**Published:** 2016-09-27

**Authors:** Mahnaz Abbasi, Mohammadali Zohal, Banafsheh Atapour, Zohreh Yazdi

**Affiliations:** ^1^Rheumatology, Metabolic Diseases Research Center, Qazvin University of Medical Sciences, Qazvin, Iran; ^2^Pulmonary Diseases, Metabolic Diseases Research Center, Qazvin University of Medical Sciences, Qazvin, Iran; ^3^Metabolic Diseases Research Center, Qazvin University of Medical Sciences, Qazvin, Iran; ^4^Occupational Medicine, Social Determinants of Health Research Center, Qazvin University of Medical Sciences, Qazvin, Iran

## Abstract

*Introduction*. Chronic obstructive pulmonary disease (COPD) is a major cause of morbidity and mortality worldwide. Proper diagnosis of osteoporosis as a systemic adverse effect of COPD is of significant importance. The present study aimed at evaluating the prevalence of osteoporosis and its risk factors in men suffering from COPD in Qazvin (2014).* Methods*. This descriptive-analytical study was conducted on 90 patients with COPD using random sampling. Anthropometric data and results from physical examination were collected. Pulmonary function test and bone mineral densitometry were done for all participants as well.* Results.* The prevalence of osteopenia and osteoporosis in COPD patients was 31.5 and 52.8 percent, respectively. Bone mineral density (BMD) at the femoral neck was associated significantly with body mass index (BMI), increased severity of COPD, and use of oral corticosteroid (*P* < 0.05).* Conclusion.* The results showed that patients' BMI and severity of COPD are two valuable risk factors for osteoporosis screening in COPD patients.

## 1. Introduction

Chronic obstructive pulmonary disease (COPD) is a progressive lung disease with prevalence rate of 5–13 percent [1, 2]. COPD is characterized by inflammation in the lung parenchyma and airways and irreversible obstruction in airflow. Patients suffering from COPD present with shortness of breath, dyspnea on exertion, cough, and sputum production [3]. It is a multisystem disorder that affects many organs such as bones causing osteopenia and osteoporosis [4, 5].

Osteopenia is a condition in which bone mineral density is reduced. Likewise, osteoporosis is a more severe condition, where decreased bone density increases the risk of fracture. The most common sites of fracture are wrist, spine, ribs, and hip. These fractures can result in pain, disability, reduced mobility, and other serious injuries [6].

Recent evidences suggest that osteoporosis in COPD patients is debilitating. Osteoporosis can cause multiple vertebral fractures in spine which result in a loss of at least 15–20% of its height. These fractures often result in patients' height loss, chronic pain, and kyphosis which ultimately damage respiratory function. The complications can lead to death if they are left untreated [7, 8].

Researchers are investigating further to clarify the relationship between osteoporosis and COPD. Most researchers, however, believe that the two diseases have a number of risk factors in common including smoking, older age, low level of vitamin D, long-term treatment with corticosteroids, excessive weight loss, and low body mass index in patients. Moreover, 22 to 69 percent of men with COPD may develop hypogonadism that has been associated with other systemic manifestations including osteoporosis and depression [4, 9, 10].

Since osteoporosis is a chronic disease similar to COPD and tends to deteriorate with age, it is crucial to pay more attention to its risk factors so as to enable its prevention, early diagnosis, and treatment. Thus, estimating the prevalence of risk factors for osteoporosis in patients with COPD is important. For example, it is possible to use bisphosphonates for prophylaxis and treatment of osteoporosis, if corticosteroids are recognized as the main culprit in COPD patients [11, 12]. Proper protective effect of bisphosphonates has been reported in glucocorticoid-induced osteoporosis in previous studies [11–13].

Since most COPD patients are old men with risk factors for osteoporosis, the present study aimed to evaluate the prevalence of osteoporosis and detect its risk factors in men with COPD in Qazvin (2014).

## 2. Methods

This cross-sectional study was performed in lung clinic of Bouali Hospital in Qazvin in 2014. Ninety men with COPD aged from 60 to 86 years were enrolled. The study was approved by the ethics committee of Qazvin University of Medical Sciences (QUMS). Inclusion criteria were diagnosis of COPD by pulmonologist and obtaining informed consent from patients, whereas exclusion criteria included history of asthma or any chronic pulmonary disorders except COPD, history of bone disease, and patients who had been treated for osteoporosis or used diuretics. In addition, patients with chronic diseases affecting the bone mineral density (e.g., hyperthyroidism) or patients with a history of thoracic surgery and malignancy were excluded. Diagnosis of COPD in the patients was considered according to criteria provided by American Thoracic Society (ATS) and European Respiratory Society (ERS) [14].

For this purpose, pulmonologist used the patients' medical records, current symptoms, and the results of pulmonary function test. Lung function was assessed by trained technicians (SPM300 spirometer). The forced expiratory volume 1 (FEV1) and forced vital capacity (FVC) were measured by spirometer, and the FEV1/FVC ratio was calculated. COPD was diagnosed for the patients through spirometry, where postbronchodilatory FEV1/FVC was lower than 70%.

Using FEV1, according to GOLD guideline, the COPD patients were then classified into four groups of severity [15].

Having calculated the body mass index (BMI) by dividing weight (in kilograms) by the square of the weight, the patients were categorized as underweight (BMI < 17 kg/m^2^), normal weight (17–25 kg/m^2^), overweight (25.1–30 kg/m^2^), and obese (>30 kg/m^2^) [16].

The bone mineral density was measured by using dual-energy X-ray absorptiometry at lumbar spine and femoral neck (Hologic QDR 2000, Bedford, MA, USA, model). The results of BMD were categorized according to the WHO criteria. Therefore, subjects with spine or femur neck *T*-score of −2.5 or below were considered as having osteoporosis. Subjects with *T*-score between −1 and −2.5 and more than −1 were considered as having osteopenia and normal people, respectively [17].

Data were presented using frequency and percentage for categorical variables. Chi-square test was used to compare qualitative variables. All statistical analyses were performed using SPSS software version 19 and *P* values less than 0.05 were considered statistically significant.

## 3. Results

A total of 90 patients with COPD aged from 60 to 86 years (mean age: 69 ± 6) participated in this study. The majority of patients (61.8%) were between 60 and 69 years, and 30.3% and 7.9% of patients were between 70 and 79 years and between 80 and 89 years, respectively. The mean body mass index (BMI) was 22.2 ± 4.17 kg/m^2^, and 44.9% of patients had normal BMI.

Based on GOLD criteria, 14 (15.7%) patients had GOLD II, 27 (30.3%) patients had GOLD III, and 31 (34.8%) patients had GOLD IV. The mean lumbar spine and neck of femur BMD of the patients were 0.85 ± 0.15 and 0.7 ± 0.12, respectively.

According to WHO criteria, 47 patients (52.8%) had osteoporosis. Results for relationship between variables and bone mineral density in the lumbar spine and neck of femur are shown in Table 1.

**Figure 1:**
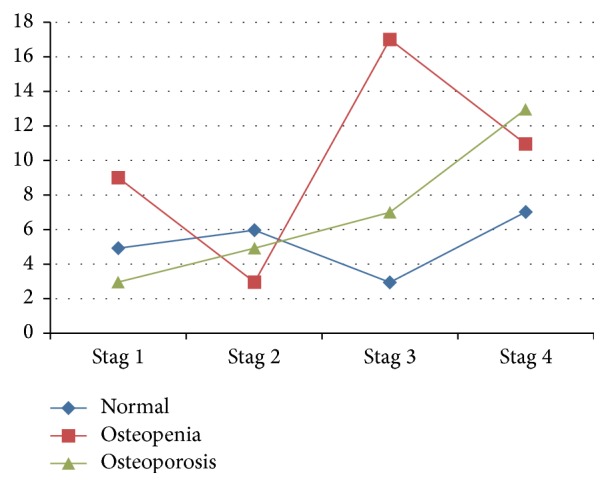
Interaction between osteoporosis in the femoral neck and severity of COPD.

As shown in the table, only BMI and severity of COPD were significantly associated with osteoporosis. This relationship was not found in other risk factors. The statistically significant relationship was observed only between severity of COPD and osteoporosis in neck of femur (*P* = 0.042). As seen in Figure 1, with increasing the severity of COPD in patients, severity of bone loss (osteoporosis) increased significantly.

**Figure 2:**
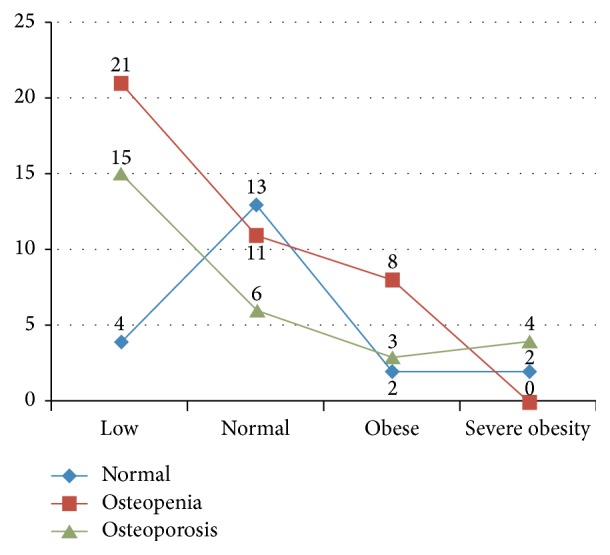
Interaction between osteoporosis in the femoral neck and patients' BMI.

Figure 2 shows the relationship between patients' BMI and osteoporosis based on *T*-score of femoral neck. As illustrated in Figure 2, reduction in patients' BMI leads to significant increase in osteoporosis.

Statistical analyses did not show any significant relationship between oral corticosteroid use and osteoporosis based on lumbar spine *T*-score. Other results, however, showed that there was a significant correlation between dose of oral corticosteroid and patients' osteoporosis based on femoral neck *T*-score (*r* = 0.26; *P* value = 0.015).

## 4. Discussion

In this study, the prevalence of osteopenia and osteoporosis in COPD patients was 31.5% and 52.8%, respectively. In a study conducted by Abu-Bakr and his colleagues, the prevalence of osteopenia and osteoporosis was observed in 50 and 30 percent of COPD patients [8]. The results were consistent with those of another study conducted by Mansour and his colleagues. They reported the prevalence of osteoporosis and osteopenia in COPD patients as a range of 9–69% and a range of 27–76%, respectively. Their explanation for the differences observed in the studies was using different diagnostic methods, different study population, and different severity in the underlying respiratory disease [8, 18].

In the present study, severity of osteoporosis according to femoral neck *T*-score increased with the increase of the severity of COPD. In other words, reduction of FEV1 in subjects was associated with low levels of bone mineral density. Nuti and his colleagues also found similar results and showed that bone mineral density in COPD patients was low and decreased with increasing disease severity [19]. Nevertheless, the relationship between lung function parameters and osteoporosis is complex and ambiguous. Cellular microstructural defects in COPD are not well understood as well [20].

An experimental study of transbone biopsy in COPD patients showed that microstructure skeletal disorders (both cancellous and cortical bones) are more in COPD patients compared to control groups [21]. It is very likely that an underlying reason for the disorders is the increased levels of cytokines and production of catalytic enzymes. It has been shown that there is a continuous systemic inflammatory state in patients with COPD, which results in the release of inflammatory cytokines such as TNF-*α*, IL-1, and IL-6. On the other hand, production of catalytic enzymes, for example, matrix metalloproteinases, increases during the course of COPD. Protein catabolic process and the inflammatory cytokines induce bone resorption and inhibit bone formation [21–24].

The use of corticosteroids in these patients is also considered as another cause of low bone density [22, 25]. This was examined in the present study as well, and a significant relationship was observed between the osteoporosis in femoral neck based on *T*-score and the use of oral corticosteroid. In other words, by increasing the oral corticosteroid use, an increased risk for osteoporosis was observed. Almost all patients were given inhaled corticosteroids in the present study, and no significant relationship was observed between the osteoporosis in lumbar spine based on *T*-score and the use of oral corticosteroid. Similarly, no association was found between inhaled corticosteroid and budesonide use with osteoporosis in both regions. The treatment, however, can be strongly confusing in the interpretation of the results concerning the relevance of COPD and low BMI (at least in this specific sample) as a risk factor for osteoporosis. Israel et al. found a reduced bone density after long-term treatment with triamcinolone [26], but studies on the effects of budesonide and fluticasone did not eventuate in the same results. In the TORCH study carried out by Ferguson et al., no significant effect on bone mineral density for inhaled corticosteroids was found [27]. In another study by Johnell et al., effects of long-term treatment with budesonide on fracture rates and bone mineral density were not found to be clinically significant [28]. In contrast, Nuti et al. showed that both severity of COPD and using inhaled oral glucocorticoid therapy increase the risk of vertebral fractures [19]. Casadp and his colleagues also found that patient's treatment with glucocorticoid and severity of COPD are correlated [29]. In a similar study, Dam et al. found that patients with COPD or asthma using corticosteroid had the lowest amount of BMD, and the risk of osteoporosis in their bones was two times more compared to those without COPD and asthma [30].

In this study, a significant relationship was found between BMI and osteoporosis in femoral neck based on *T*-score, such that a reduction in BMI was shown to be associated with increased severity of osteoporosis. However, no association was observed between BMI and osteoporosis based on *T*-score of lumbar spine. Bone mass is directly associated with BMI and patients with a higher BMI have higher BMD [31, 32]. This is partly due to the effect of weight bearing on bones and the higher levels of estrogen in obese people. Aromatization and the conversion of testosterone to estrogen increase in obese people [33]. The effect of BMI on BMD is reportedly dependent on the bone place in body. Accordingly, hip would be the first to be affected followed by lumbar spine and distal radius, respectively. It has also been suggested that this order may be due to differences in body fat percentage and weight bearing condition in different areas [34]. The mechanism of the relationship between low BMI and osteoporosis is not fully clear in patients with COPD. It is probably due to the increase in inflammatory processes, the decrease in physical activity, and other mechanisms of proteolysis [35–38]. Therefore, osteoporosis is considered as a major problem in men with COPD. It can cause multiple fractures in spines and reduce forced vital capacity (FVC) in COPD patients and, consequently, can exacerbate the adverse effects of COPD in patients. Therefore, identifying and controlling the common risk factors for COPD and osteoporosis are essential in any society [8].

## 5. Conclusion

The results showed that an increase in severity of COPD, using oral corticosteroid, and decrease of BMI are three risk factors for high prevalence of osteoporosis in COPD patients in Qazvin province. Given the importance of osteoporosis in COPD, it is recommended that all COPD patients be screened for osteoporosis so as to prevent serious complications such as but not limited to fractures.

## Figures and Tables

**Table 1:** Frequency of different risk factors of osteoporosis in COPD patients.

Variable	Spine			*P* value	Femur			*P* value
	Normal	Osteopenia	Osteoporosis		Normal	Osteopenia	Osteoporosis	
Age (year)								
60–69	10 (11.2%)	19 (21.3%)	26 (29.2%)	0.49	13 (14.6%)	28 (31.5%)	14 (15.7%)	0.52
70–79	8 (9%)	8 (9%)	11 (12.4%)		7 (7.9%)	9 (10.1%)	11 (12.4%)	
80–89	0 (0%)	3 (3.4%)	4 (4.5%)		1 (1.1%)	3 (3.4%)	3 (3.4%)	
Smoking (pack/year)								
<20	2 (2.2%)	8 (9%)	8 (9%)	0.76	5 (5.6%)	9 (10.1%)	4 (4.5%)	0.82
21–40	7 (7.9%)	10 (11.2%)	16 (18%)		7 (7.9%)	16 (18%)	10 (11.2%)	
>40	9 (10.1%)	12 (13.5%)	17 (19.1%)		9 (10.1%)	15 (16.9%)	14 (15.7%)	
Severity of COPD								
Mild (grade I)	6 (6.7%)	4 (4.5%)	7 (7.9%)	0.38	5 (5.6%)	9 (10.1%)	3 (3.4%)	0.042
Moderate (grade II)	1 (1.1%)	6 (6.7%)	7 (7.9%)		6 (6.7%)	3 (3.4%)	5 (5.6%)	
Severe (grade III)	3 (3.4%)	11 (12.4%)	13 (14.6%)		3 (3.4%)	17 (19.1%)	7 (7.9%)	
Very severe (grade IV)	8 (9%)	9 (10.1%)	14 (15.7%)		7 (7.9%)	11 (12.4%)	13 (14.6%)	
BMI								
Low	19 (21.3%)	13 (14.6%)	8 (9%)	0.79	4 (4.5%)	21 (23.6%)	15 (16.9%)	0.034
Moderate	10 (11.2%)	15 (16.9%)	5 (5.6%)		13 (14.6%)	11 (12.4%)	6 (6.7%)	
Overweight	8 (9%)	3 (3.4%)	2 (2.2%)		2 (2.2%)	8 (9%)	3 (3.4%)	
Obese	2 (2.2%)	4 (4.5%)	0 (0%)		2 (2.2%)	0 (0%)	4 (4.5%)	
Corticosteroid								
Budesonide	2 (2.2%)	4 (4.5%)	4 (4.5%)	0.89	3 (3.4%)	5 (5.6%)	2 (2.2%)	0.69
Inhaled corticosteroid	17 (19.1%)	30 (33.7%)	40 (44.9%)	0.45	20 (22.5%)	40 (44.9%)	27 (30.3%)	0.42
